# Study on the antibacterial effect of the new anti-biofilm inhibitor ICAC on *Escherichia coli*

**DOI:** 10.3389/fcimb.2025.1710407

**Published:** 2025-12-10

**Authors:** Yubin Bai, Zixuan Shang, Rongbin Hu, Xun Gao, Zhijin Zhang, Bing Li, Zhen Zhu, Jiyu Zhang

**Affiliations:** 1 Key Laboratory of New Animal Drug Project of Gansu Province, Key Laboratory of Veterinary Pharmaceutical Development of the Ministry of Agriculture, Lanzhou Institute of Husbandry and Pharmaceutical Sciences of Chinese Academy of Agricultural Sciences (CAAS), Jiangouyan, Lanzhou, China; 2 School of Life Science and Food Engineering, Hebei University of Engineering, Handan, China

**Keywords:** Stevia rebaudiana isochlorogenic acid C, *Escherichia coli*, biofilm, EPS, motility, c-di-GMP

## Abstract

The rise of bacteria antibiotics resistance has bacome increasingly severe, and the development of natural compounds with antibacterial activity represents a promising approach to combat this issue. The efficacy and mechanisms of the plant-derived phenolic compound isochlorogenic acid C (ICAC) as an antibacterial and antibiofilm agent against *E. coli* were investigated. The study utilized a comprehensive approach encompassing crystal violet staining, scanning electron microscopy (SEM), confocal laser scanning microscopy (CLSM), the ruthenium red method, semi-solid agar motility assays, and quantitative real-time PCR (qRT-PCR) to elucidate the inhibitory effects and their underlying mechanisms. Results revealed that ICAC exhibited significant antibacterial and antibiofilm activity against *E. coli*. The results demonstrated that ICAC could inhibit the biofilm formation of *E. coli*, reduce the biomass of preformed biofilms, and decrease the production of extracellular polysaccharides (EPS) and extracellular proteins, as well as bacterial motility. Moreover, qRT-PCR results showed that ICAC downregulated genes associated with c-di-GMP synthesis while upregulating those involves in c-di-GMP degradation, thereby inhibiting biofilm formation and bacterial motility. In summary, ICAC shows potential as an effective anti-c-di-GMP agent and a novel antibacterial candidate for the treatment of E. coli infections.

## 1 Introduction

Antibiotic resistance has been an increasingly severe global public health issue. The World Health Organization estimates that by 2050, approximately 10 million people deaths per year will be attributable to antimicrobial resistance, with associated economic losses exceeding 100 trillion US dollars (Defoirdt, 2025). The horizontal transfer of antibiotic resistance genes facilitates the spread of antibiotic resistance to human pathogens, which can lead to the emergence of antibiotic-resistant superbugs that pose a direct threat to human health (Li et al., 2025). As antibiotic resistance escalates into a global health crisis, there is an urgent need for novel therapeutic approaches to bacterial diseases (Wu et al., 2025). A biofilm is a microbial community that adheres to surface and is encased within a self-produced extracellular matrix (Solano et al, 2014). A study of biofilm bacteria demonstrated that they could withstand antibiotic concentrations more than 1000 times greater than those tolerated by planktonic bacteria (Sharma et al, 2019). Biofilms are involved in approximately two-thirds of bacterial infections (Cui et al, 2022). These biofilm-associated infections are closely linked to the bacteria resistance, virulence and pathogenicity. Therefore, targeting bacterial biofilms has emerged as an effective strategy to combat bacterial resistance and infection (Wang et al, 2022).

Plant extracts are natural substances with wide available, and contain active small molecular components that have been extensively utilized in the treatment of various diseases (Li et al, 2022). Plants produces a diverse array of secondary metabolites to interact with their environment, some of which modulate local microbial communities and biofilms formation by influencing the growth and function of bacteria (Dettweiler et al, 2019). Ginkgetin reduces biofilm formation, exopolysaccharide (EPS) production, motility in *E. coli*, while significantly enhance its sensitivity to colistin antibiotics without inhibiting bacterial growth and metabolic activity (Bai et al, 2022). Chlorogenic acid, a phenolic compound from the hydroxycinnamic acid family, exhibits a wide range of biological activities, including antibacterial, antioxidant, and anti-inflammatory effects (Naveed et al, 2018). Wang also found that chlorogenic acid significantly inhibits the formation, aggregation, and virulence factors of biofilms (Wang et al, 2019). Isochlorogenic acid C (ICAC), a derivative of chlorogenic acid, has been widely reports to possess antioxidant, anti-inflammatory, and cardiovascular protective effects (Wu et al., 2025). Although chlorogenic acid and its derivatives have shown various biological activities, the molecular pathway through which ICAC regulates Escherichia coli biofilm via c-di-GMP signaling remains to be explored. This study aimed to evaluate the antibacterial and antibiofilm effects of ICAC against *E. coli* through *in vitro* assays combining microscopy, metabolic, and gene expression analyses, in order to elucidate its regulatory mechanism on c-di-GMP signaling and assess its potential as a natural antibiofilm agent for controlling bacterial resistance. The findings provide a foundation for the potential application of ICAC as a natural compound in addressing bacterial resistance. The work suggests promising prospects for the development of antimicrobial agents and strategies for controlling bacterial resistance.

## 2 Materials and methods

### 2.1 Bacterial strains and chemicals

Clinically isolated *E. coli A4E1*, along with additional biofilm-producing strains isolated from livestock and poultry manure in cattle, pig, and chicken farms in Hebei region, were provided by the College of Life Sciences and Food Engineering at Hebei University of Engineering. These strains were cultivated using Luria-Bertani (LB, HuanKai Microbial, Guangdong, China) and Luria-Bertani agar (LA, HuanKai Microbial, Guangdong, China) medium. Isochlorogenic acid C (ICAC, purity 94.2%, HPLC identification) from stevia, sourced from Chenguang Biotechnology Co., Ltd. ICAC was dissolved in a solution composed of 20% DMSO, 10% Tween 80, 30% PEG300, and 40% water to obtain a final concentration of 64 mg/ml. Crystal violet from Solarbio (Solarbio Science & Technology Co., Ltd., Beijing, China), with all other reagents being of analytical grade.

### 2.2 Biofilm formation capacity assay

With slight modifications to the previous method, biofilms formation was assessed using CV staining according to Cho JA (Cho et al, 2022). Briefly, the 200μl suspension of *E. coli* (OD600 = 0.01) was inoculated into a 96-well plate (Costar 3599, *Corning* Inc) and incubated statically at 37°C for 24 h. After incubation, the wells was washed three times with 300μl of PBS (pH = 7.2), and fixed with 150μl of methanol at room temperature for 1 hour. The biofilm was stained with 150μl of 0.1% CV for 30 minutes, followed by rinsing with distilled water and drying at 55°C. The bound CV was then dissolved in 150μl of 95% ethanol, and measure the absorbance at 570 nm. The results are categorized based on the ODc value, where ODc equals the mean of the negative control wells plus three times its standard deviation: non-biofilm-forming strains (OD ≤ ODc), weak biofilm-forming strains (ODc < OD ≤ 2ODc), moderate biofilm-forming strains (2ODc < OD ≤ 4ODc), and strong biofilm-forming strains (4ODc < OD). During the experiment, blank wells were used to correct background absorbance, and all data were calculated in per plate. Three biological replicates were set up and all experiments were repeated 3 times.

### 2.3 Determination of biofilm maturation time

According to previous described methods (Deng et al, 2021), the 200μl suspension of *E. coli* (OD600 = 0.01) was inoculated into a 96-well plate (Costar 3599, Corning Inc), using LB nutrient broth as the blank control, and incubated statically at 37°C. The LB nutrient broth was replaced daily. At 12 h, 1 d, 2 d, 3 d, 4 d, 5 d, 6 d, and 7 d, planktonic bacteria were removed by washing with sterilized saline, followed by crystal violet staining. The OD570nm was measured, and the biofilm growth curve was plotted based on the OD values. Three biological replicates were set up and all experiments were repeated 3 times.

### 2.4 Determination of minimum inhibitory concentration *in vitro* and growth curve

The MICs of the compounds used in this study were determined using to the broth microdilution method (Pezeshkpour et al, 2018). Briefly, bacteria were cultured in MHB medium at 37°C with shaking at 150rpm and adjusted to a 0.5 McFarland standard, followed by a 1: 100-fold dilution. In a 96-well cell culture plate (Costar 3599, Corning Inc), serial dilutions of ICAC were added to each well containing 200 µL of the diluted bacterial suspension, and the plate was incubated statically at 37°C for 16 to 18 hours. Each experiment included a bacterial control well (bacteria without ICAC) and a growth control well (Only medium). The MIC value was defined as the lowest concentration that completely inhibited bacterial growth by visual turbidity. The growth curves of A4E1 under different concentrations of ICAC were plotted with slight modifications to the previous method (Yue et al, 2018). ICAC was added at concentrations of 1/2 MIC, 1/4 MIC, and 1/8 MIC, and the cultures were incubated in a 37°C with shaking at 150 rpm. the OD600 values was measured every 2 h for 24h using a microplate reader. In all experiment, DMSO was employed as the control group, and the OD values were normalized relative to the untreated control. Three biological replicates were set up and all experiments were repeated 3 times.

### 2.5 Measurement of metabolic activity

According to previous methods, the metabolic activity of *E. coli* was analyzed using the Alamar Blue (AB) assay (Bai et al, 2022). *E. coli* A4E1 was cultured overnight, diluted to OD600 = 0.01, and then mixed with different concentrations of ICAC. The mixtures were inoculated into a 48-well plate (*Corning*, NY, USA) at 37°C for 24 h. The changes in growth metabolic activity were measured using the Alamar Blue assay. Cells from each well were collected, centrifuged at 10000 rpm for 5 min, washed twice with sterile PBS (pH=7.4), and resuspended. PBS containing only AB dye was used as a blank control, and the absorbance at 570 nm and 600 nm was determined using the following formula. Metabolic activity(%)= ( (Eoxi (OD570) × TOD570)- (Eoxi (OD600) × TOD600))/ ( (Ered (OD570) × BOD570)- (Ered (OD600) × BOD600))×100%, Eoxi (OD570)-extinction coefficient in oxidized form of AB at 570nm = 80586, Ered (OD570)-extinction coefficient in reduced form of AB at 570nm = 155677, Eoxi (OD600)-extinction coefficient in oxidized form of AB at 600nm = 117216, Ered (OD600)-extinction coefficient in reduced form of AB at 570nm = 14652, B-blank, T-samples. Three biological replicates were set up and all experiments were repeated 3 times.

### 2.6 ICAC inhibits biofilm formation

According to the previous methods (Zuo et al, 2019), the 200μl suspension of bacterial and drugs were co-inoculated into a 96-well plate (Costar 3599, Corning Inc) to obtain final ICAC concentrations of 1/2 MIC, 1/4 MIC, 1/8 MIC, and 1/16 MIC, with an untreated control group included. The plate was incubated statically at 37°C for 24 hours. After incubation, the wells was washed three times with 300μl of PBS (pH = 7.2), and fixed with 150μl of methanol at room temperature for 1 hour. The biofilm was stained with 150μl of 0.1% CV for 30 minutes, followed by rinsing with distilled water and drying at 55°C. The bound CV was then dissolved in 150μl of 95% ethanol, and measure the absorbance at 570 nm. Three biological replicates were set up and all experiments were repeated 3 times.

### 2.7 The impact of ICAC on preformed biofilms of *E. coli*

According to the established method (Chen et al, 2022), the 200μl suspension of bacterial as inoculated into a 96-well plate (Costar 3599, Corning Inc) and incubated statically at 37°C for 24 hours. After removing and discarding the supernatant, each well was washed with 300μl of PBS. Drugs were then added to achieve final concentrations of 2MIC, 1MIC, 1/2 MIC, and 1/4MIC, while the control group remained untreated. The plate was further incubated statically at 37°C for 24 hours. After incubation, the wells was washed three times with 300μl of PBS (pH = 7.2), and fixed with 150μl of methanol at room temperature for 1 hour. The biofilm was stained with 150μl of 0.1% CV for 30 minutes, followed by rinsing with distilled water and drying at 55°C. The bound CV was then dissolved in 150μl of 95% ethanol, and measure the absorbance at 570 nm. Three biological replicates were set up and all experiments were repeated 3 times.

### 2.8 EPS production

According to the previously described method, EPS production was assessed by ruthenium red staining (Adnan et al, 2020). Briefly, 200μl suspension of *E. coli* A4E1 (OD_600_ = 0.01) was mixed with different concentrations of ICAC solution and dispensed to a 96-well plate (Costar 3599, Corning Inc). After static incubation at 37°C for 24 hours, the culture medium was removed, and each well was washed with 300μl of PBS. Subsequently, 150μl of 0.01% ruthenium red solution was added to each well. Wells containing only ruthenium red solution served as the blank control, while wells with biofilm untreated by ICAC but stained with ruthenium red were used as the positive control. The plate was incubated at 37°C for 60 minutes, after which the liquid containing residual stain was transferred to a new 96-well plate. Absorbance was measured at 450 nm. The inhibition rate was calculated using the following formula: EPS inhibition (%)= ( (AS-AP)/ (AB-AP))×100%, AB: Absorbance of the blank control solution, AS: Absorbance of the sample solution, AP: Absorbance of the positive control solution. Three biological replicates were set up and all experiments were repeated 3 times.

### 2.9 Determination of extracellular proteins in biofilms

According to previous methods, the extracellular proteins content in biofilms was determined using the Bradford method (Siddique et al, 2020). The protein standard was prepared at a final concentration of 0.2 mg/mL. Aliquots of 0, 2, 4, 6, 8, 12, 16, and 20 μL of the standard were pipetted into a 96-well plate (Costar 3599, Corning Inc), and each well was brought to a total volume of 20 μL. The A4E1 bacterial solution was treated with different concentrations of ICAC solution and then transferred to the 96-well plate, followed by static incubation at 37°C for 24 hours. After appropriately dilution, and 20 μL of each sample was added to the 96-well plate. Subsequently, each well was supplemented with diluted 1×G250 dye solution and incubated at room temperature for 3–5 minutes. The A595 was measured using a microplate reader, and the protein concentration in the samples was calculated based on the standard curve. During the experiment, a drug control group (containing only the ICAC) was set up to eliminate color interference from ICAC. Three biological replicates were set up and all experiments were repeated 3 times.

### 2.10 Motor activity measurement

Based on a previous reported methods with modifications, the motility assay of *E. coli* was performed as described by Lee JH (Lee et al, 2014). Briefly, the overnight culture of *E. coli A4E1* was diluted to an OD600 = 0.01. Motility was then assessed using semi-solid agar LB media (0.3%, 0.5%, 1% agar (Solaibao Co., Ltd, Beijing)) containing 1/2 MIC, 1/4 MIC, 1/8 MIC of ICAC. 1 μL of the diluted bacterial suspension was inoculated into the center of the petri dish. DMSO alone was used as a control. After incubation at 37°C for 16 h, the diameter of the halo zone was measured by vernier caliper to assess bacterial motility. Three biological replicates were set up and all experiments were repeated 3 times.

### 2.11 Scanning electron microscopy

According to the previously described method, the effects of the ICAC on the inhibiting bacterial biofilm formation and eradicating biofilms were evaluated using a 48-well plate (*Corning*, NY, USA) equipped with polylysine coverslips (Xiong et al, 2022).

For the inhibition assay: Bacterial suspensions containing different concentrations of ICAC were prepared, with an untreated bacterial suspension serving as the control group. The bacterial suspensions were mixed with ICAC, and added to the 48-well plate containing coverslips, followed incubated at 37°C for 24 hours. After incubation, the supernatant was removed, and the coverslips were gently washed with sterile PBS buffer (pH=7.4) and air-dried. The coverslips were then fully immersed in 2.5% glutaraldehyde solution and fixed at 4°C for 12 hours, after which they were dried. Subsequent dehydration using a graded ethanol series (30%, 50%, 80%, 90%, and 100%) to the critical point. Finally, the samples were sputter-coated with gold and observed under SEM (JSM-6701F, JEOL, Japan) to assess biofilm formation. SEM was performed at an accelerating voltage of 5 kV and a magnification of × 5000.

For the preformed biofilm assay: First, bacterial suspensions were first added to a 48-well plate containing coverslips and incubated at 37°C for 24 hours. The bacterial suspensions was then removed, and the coverslips were washed with sterile PBS buffer (pH=7.4) and dried. Different concentrations of ICAC solution are prepared, with LB broth used as the control. The ICAC solutions were added to the 48-well plate, while LB broth was added to the control wells, and the plate incubated at 37°C for 24 hours. After removal of the medium, the coverslips were washed with sterile PBS buffer (pH=7.4), dried, and fixed by completely immersed in 2.5% (v/v) glutaraldehyde at 4°C for 12 hours. Following fixation and drying, the coverslips were dehydrated through an ethanol gradient (30%, 50%, 80%, 90%, and 100%) to the critical point. After gold sputtering, the biofilm eradication is observed under SEM (JSM-6701F, JEOL, Japan). SEM was performed at an accelerating voltage of 5 kV and a magnification of × 5000.

### 2.12 Confocal laser scanning microscope

The 3D architecture of the E. coli biofilm was visualized using CLSM, following a previously established protocol with minor modifications (Duarte et al, 2024). Briefly, different concentrations of ICAC (1/2 MIC, 1/4 MIC, 1/8 MIC) were added to the *E. coli A4E1* bacterial suspension (OD600 = 0.01), and inoculate into a covered 6-well plate (*Corning*, NY, USA). The plate was incubated statically at 37°C for 24 h. After incubation, the suspension were removed and the wells were washed with PBS (pH = 7.2). Biofilms were stain using the BacLight Live/Dead viability kit (L7012, Invitrogen™, Thermo Fisher Scientific, USA) following the manufacturer’s instructions. Two stock solutions of SYTO9 and PI were diluted in PBS (pH 7.2), and 500mL was added to each well at a 1 mL/3 mL/3 mL ratio. The washing holes were dried after 15 min. Live cells stained with SYTO9 (excitation wavelength at 480 nm and emission wavelength at 500 nm.) and dead cells stained with PI (excitation wavelength at 490 nm and emission wavelength at 635 nm.) were observed by CLSM (Zeiss LSM800, Carl Zeiss AG, Tokyo, Japan). The visual field was randomly selected.

### 2.13 qRT-PCR

Based on established methodologies, qRT-PCR technology was utilized to examine the effects of ICAC on the regulation of c-di-GMP, biofilm formation, and motility-related genes in *E. coli* (Bai et al, 2023). *E. coli A4E1* was cultured in 12-well plates (*Corning*, NY, USA) with and without ICAC at 37°C for 24 hours. total RNA was extracted using the modified bacterial RNA kit (AC0402, Sparkjade Biotechnology Co., Ltd, Jinan, China), and its concentration was performed using the NanoDrop OneC spectrophotometer (Thermo Scientific, USA). Reverse transcription of RNA into cDNA using the PrimeScript™ RT reagent Kit with gDNA Eraser (TAKARA Corporation, Japan). Quantitative real-time PCR analysis was carried out using TB Green^®^ Premix Ex Taq™ II (Tli RNaseH Plus) (TAKARA Corporation, Japan) in a 20 μL reaction system. Relative gene expression levels were calculated via the 2^−ΔΔCt^ method using a qPCR system (QuantStudio-6Flex machine, Applied Biosystems), with the *16s rRNA* gene serving as the internal reference gene. The reaction conditions were set as follows: initial denaturation at 95°C for 30 minutes, followed by 40 cycles of denaturation at 95°C for 5 seconds and annealing/extension at 60°C for 34 seconds. Three biological replicates were set up and all experiments were repeated 3 times. The primers used in this study are listed in **Supplementary Table S1**.

### 2.14 Statistical analysis

Statistical analysis was conducted using SPSS software (IBM, USA). Unless otherwise specified, the statistical significance of comparison was determined by one-way analysis of variance (ANOVA). Prior to ANOVA, data normality and homoscedasticity were verified. *Post hoc* analysis was conducted using Tukey’s HSD test. A *p* < 0.05 were deemed statistically significant at 95% confidence interval.

## 3 Results

### 3.1 Screening of strong biofilm-forming strains

Biofilms allow bacteria to adapt to environmental changes, and promote the development of antibiotic resistance. Without biofilms, persistent cells can survive high doses of antibiotic and evade host immune defense (Rather et al, 2021). Using the crystal violet staining method, 41 strains of *E. coli* were screened for biofilm formation: 9 strains showed strong biofilm-forming ability, 16 moderate, and 26 weak (**Supplementary Table S2**). Antibiotic susceptibility testing was conducted on the strains, and based om its strong biofilm-forming ability and high drug resistance, A4E1 was selected for further experiments. The bacterial biofilm formation capacity and antibiotic susceptibility results are summarized in **Supplementary Figure S1** and **Supplementary Table S3**.

### 3.2 Determination of biofilm maturation time

**Figure 1:**
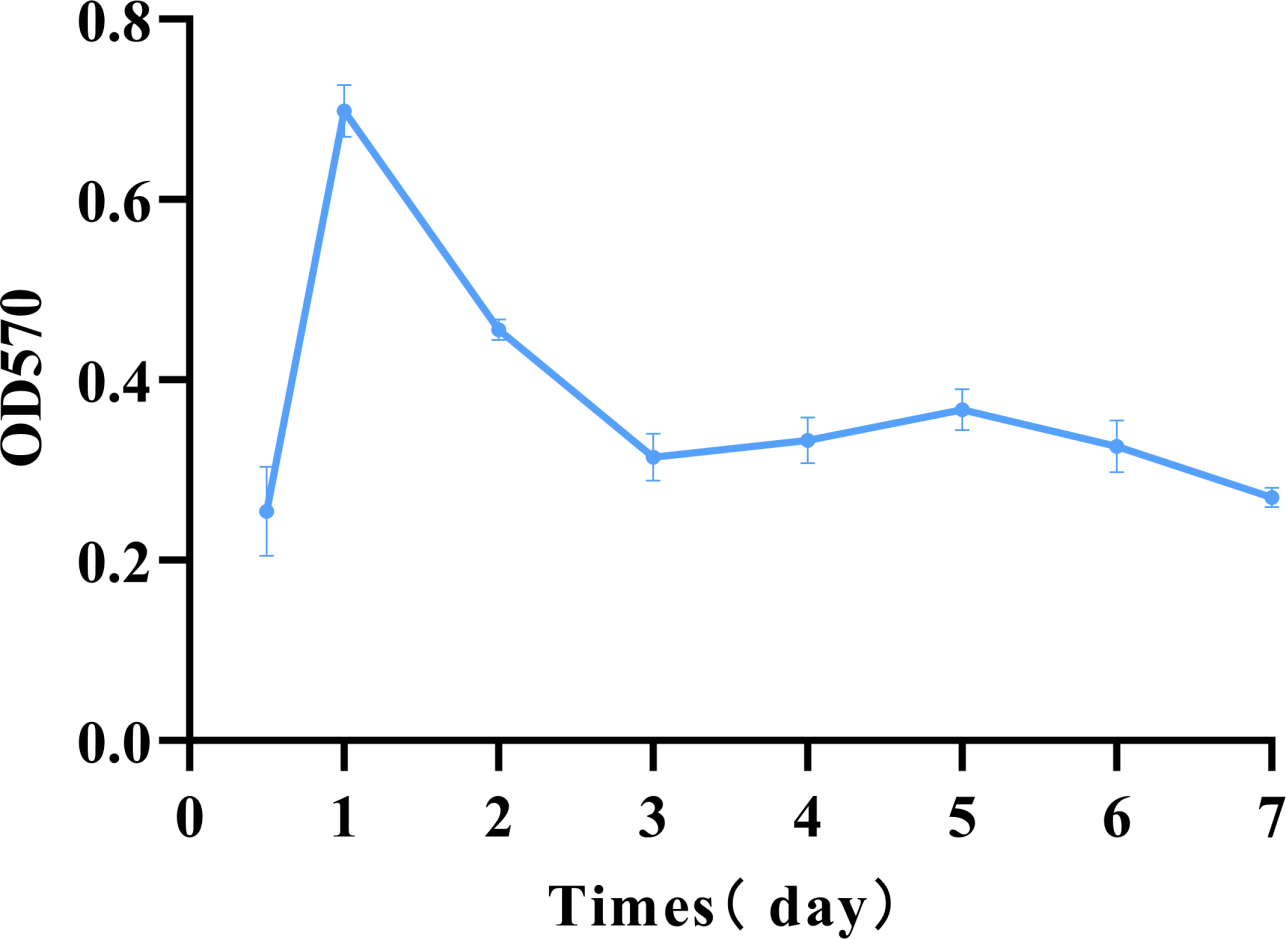
Determination of *E. coli* A4E1 biofilm maturation time. The data are presented as mean ± standard deviation.

Biofilm formation is a dynamic process comprising colonization, development, maturation, detachment, and re-colonization (Bai et al, 2023). After 12 hours of incubation, *E. coli A4E1* has adhered, and biofilm formation reached its maximum level on the first day. The mature biofilm begins to detach within 2–3 days, after which the biofilm enters the next growth cycle, as shown in **Figure 1**.

### 3.3 Determination of minimum inhibitory concentration *in vitro* and growth curve

**Figure 2:**
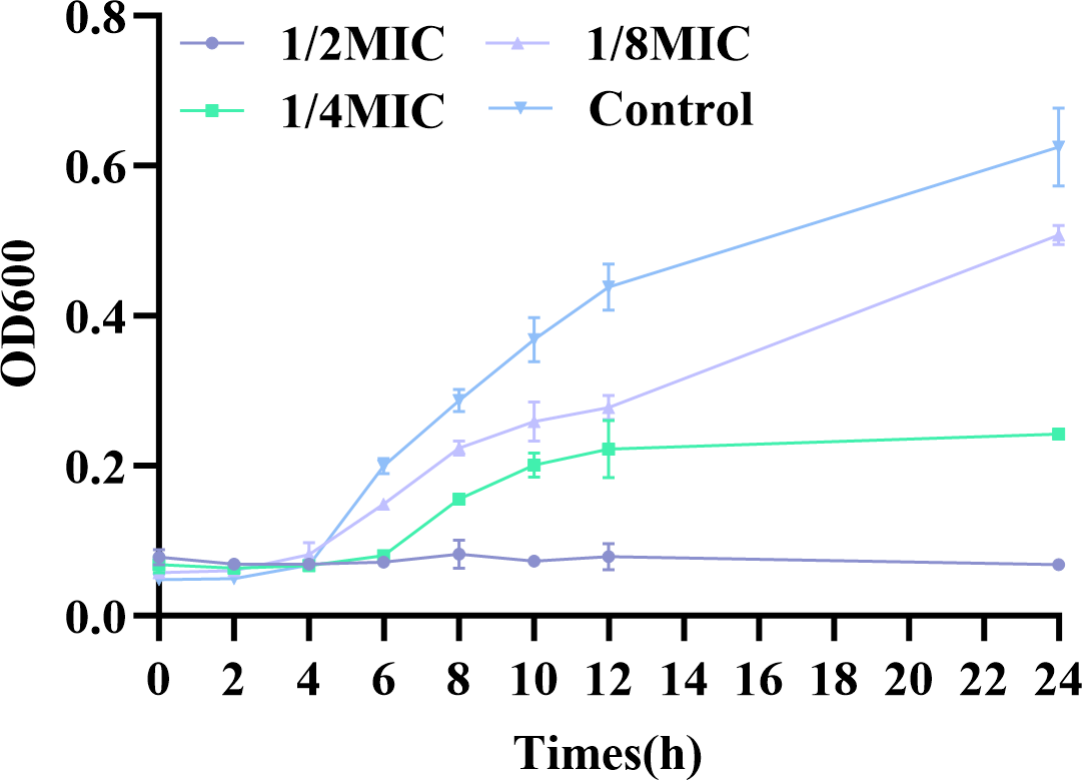
The time-dependent effect of ICAC on the growth of *E. coli* A4E1. The data are presented as mean ± standard deviation.

The minimum inhibitory concentration (MIC) of *E. coli* A4E1 was determined to be 2 mg/mL by the microbroth dilution method. Growth curve studies were performed at 1/2 MIC, 1/4 MIC, and 1/8 MIC of ICAC, as shown in **Figure 2**. The results indicated that the inhibitory effects on bacterial growth was concentration dependent, with increasing drug concentration leading to progressively stronger suppression. At the 1/8 MIC, a modest inhibitory effect on bacterial growth was observed, although it was relatively weak. In contrast, 1/4 MIC significantly suppressed bacterial growth, delaying the entry into the exponential phase and reducing the growth rate. Furthermore, 1/2 MIC exhibited strong bactericidal activity, effectively preventing bacterial proliferation.

### 3.4 Measurement of metabolic activity

**Figure 3:**
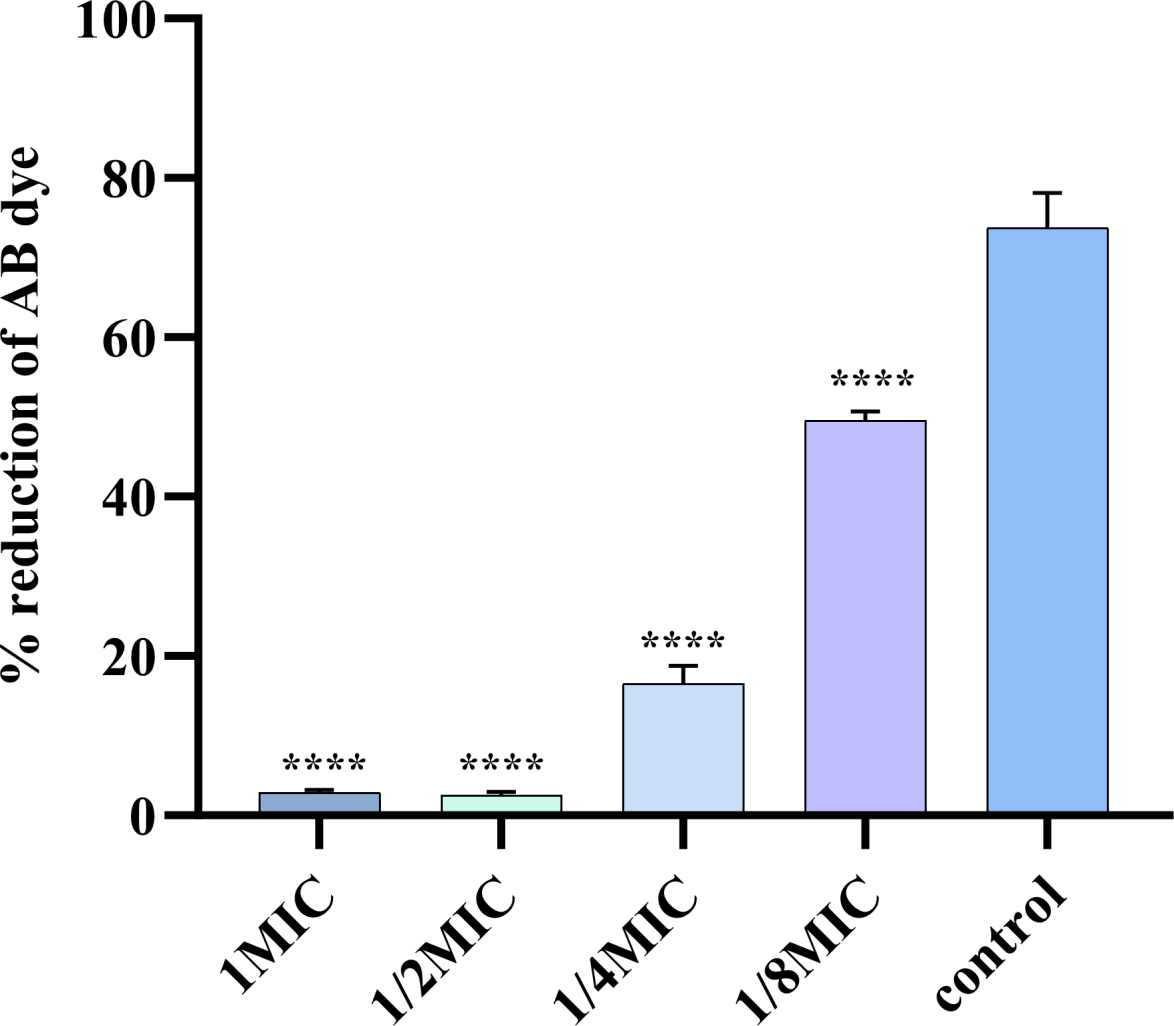
The effect of ICAC on the growth and metabolic activity of *E. coli*. The data are presented as mean ± standard deviation. ( ****P< 0.0001).

The effects of ICAC on growth and metabolic activity were assessed by the Alamar Blue (AB) assay (**Figure 3**). The results demonstrated significant differences in the fluorescence intensity of AB dye between the control group and the cells treated with ICAC. Moreover, a dose-dependent inhibition of bacterial growth and metabolic capacity with increasing concentration of ICAC.

### 3.5 ICAC’s role in biofilm inhibition and eradication

**Figure 4:**
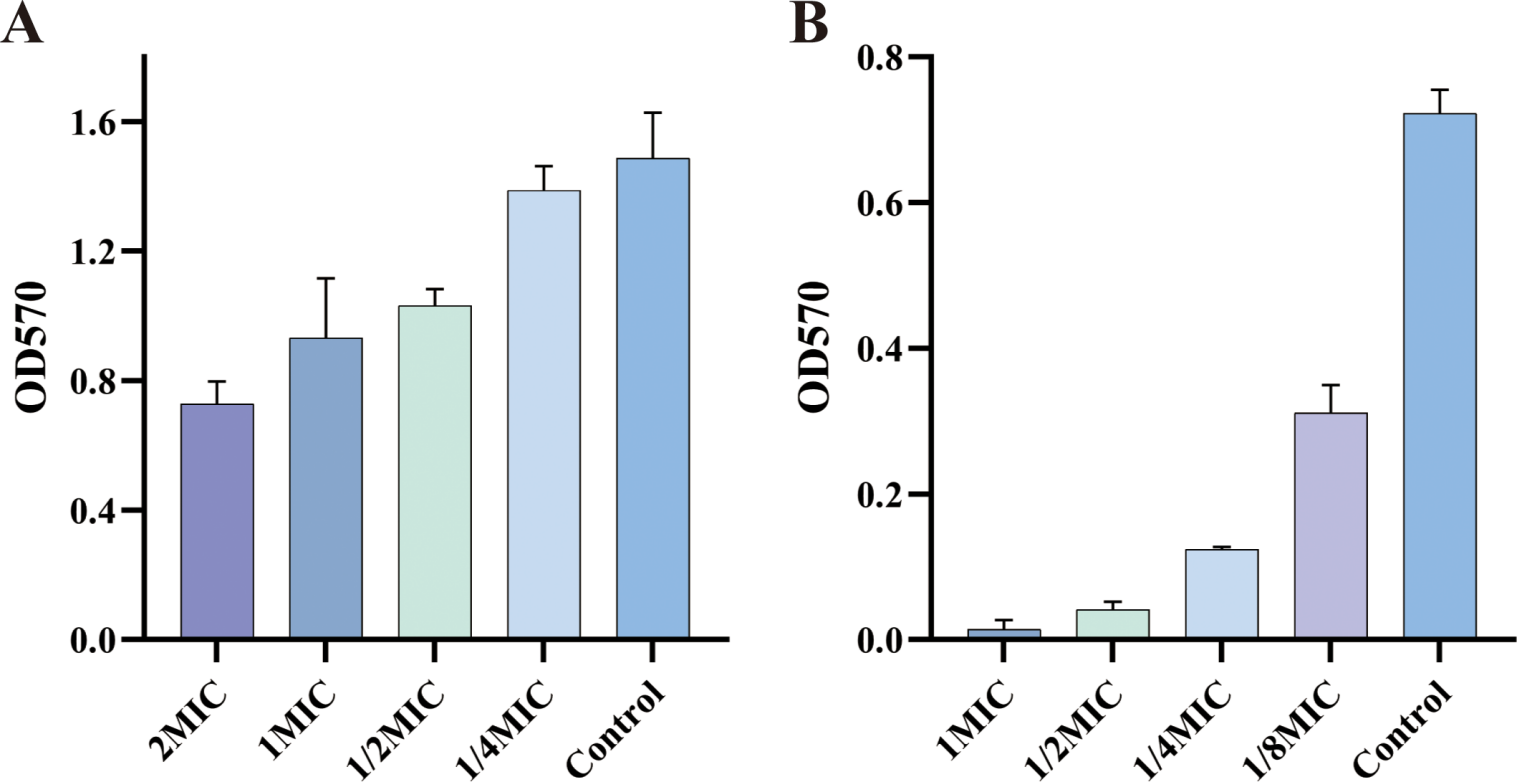
The effect of ICAC on eradicating and inhibiting *E coli* biofilms [**(A)** Eradication; **(B)** Inhibition]. The data are presented as mean ± standard deviation.

Our findings demonstrate that ICAC can significantly reduce the biomass of preformed *E. coli* biofilms in a dose-dependent manner. As the drug concentration progressively increases, the ability to eradicate biofilms is gradually enhanced, leading to a notable reduction in the adhesion of *E. coli* to polystyrene (**Figure 4A**). Additionally, ICAC can also significantly inhibit the formation of biofilms in a dose-dependent manner. With the gradual increase in drug concentration, the inhibitory effect on biofilm formation is progressively strengthened (**Figure 4B**).

### 3.6 EPS production

**Figure 5:**
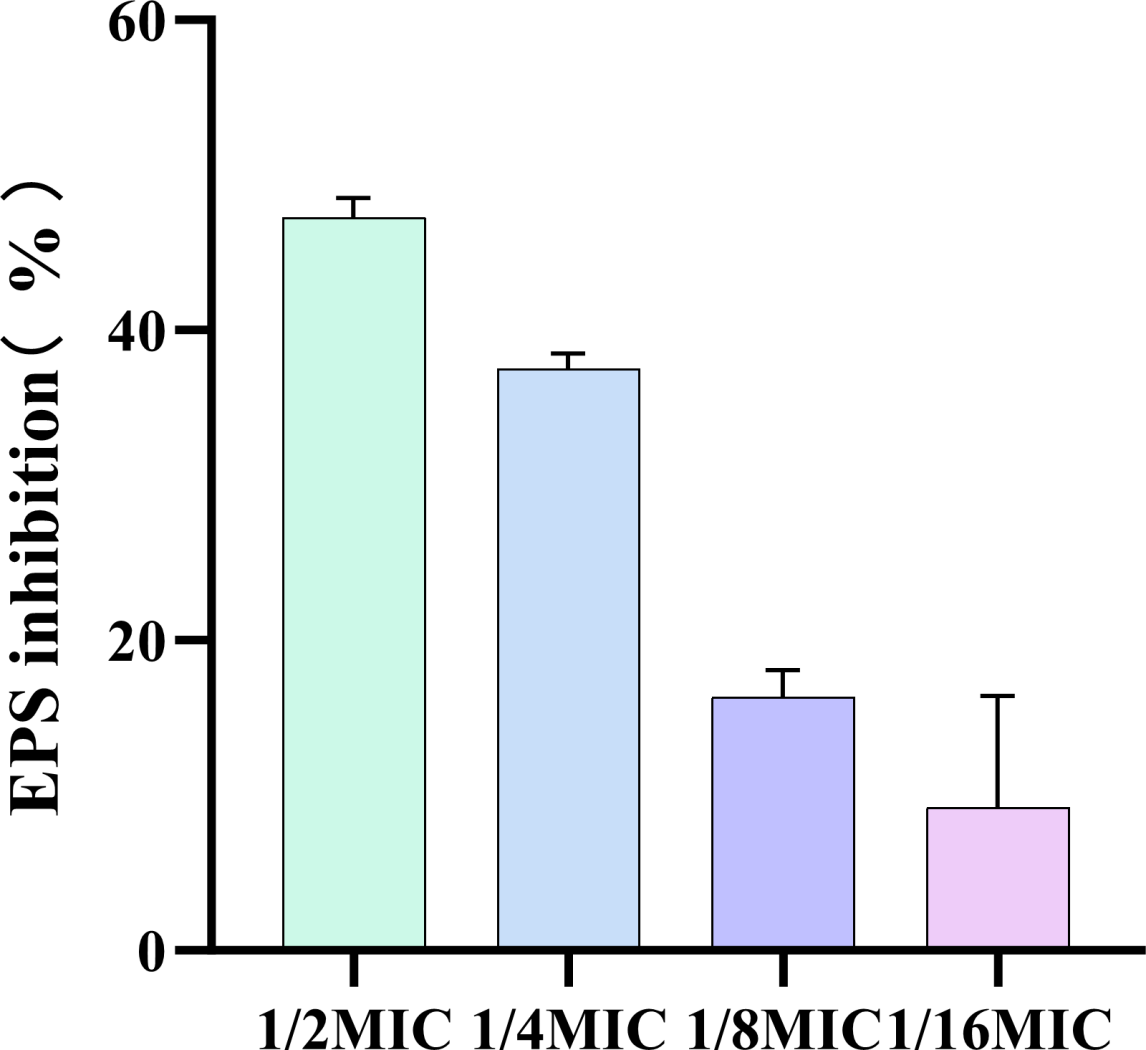
The inhibitory effect of different concentrations of ICAC on EPS production (%). The data are presented as mean ± standard deviation.

The biofilm matrix comprise secreted proteins, extracellular polysaccharides, and extracellular DNA, collectively referred to as extracellular polymeric substances (EPS) (Flemming and Wingender, 2010). EPS is considered the primary structural scaffold of biofilms and must be degraded to effectively biofilms remove (Nagaraj et al, 2018). Our finding that at a concentration of 1/2 MIC, ICAC results in a 50% inhibition rate of EPS production. The inhibition effect of ICAC on EPS production is dose-dependent, with the inhibition rate increasing progressively as the drug concentration rises, which is consistent with the CV staining results (**Figure 5**).

### 3.7 Determination of extracellular proteins in biofilms

**Figure 6:**
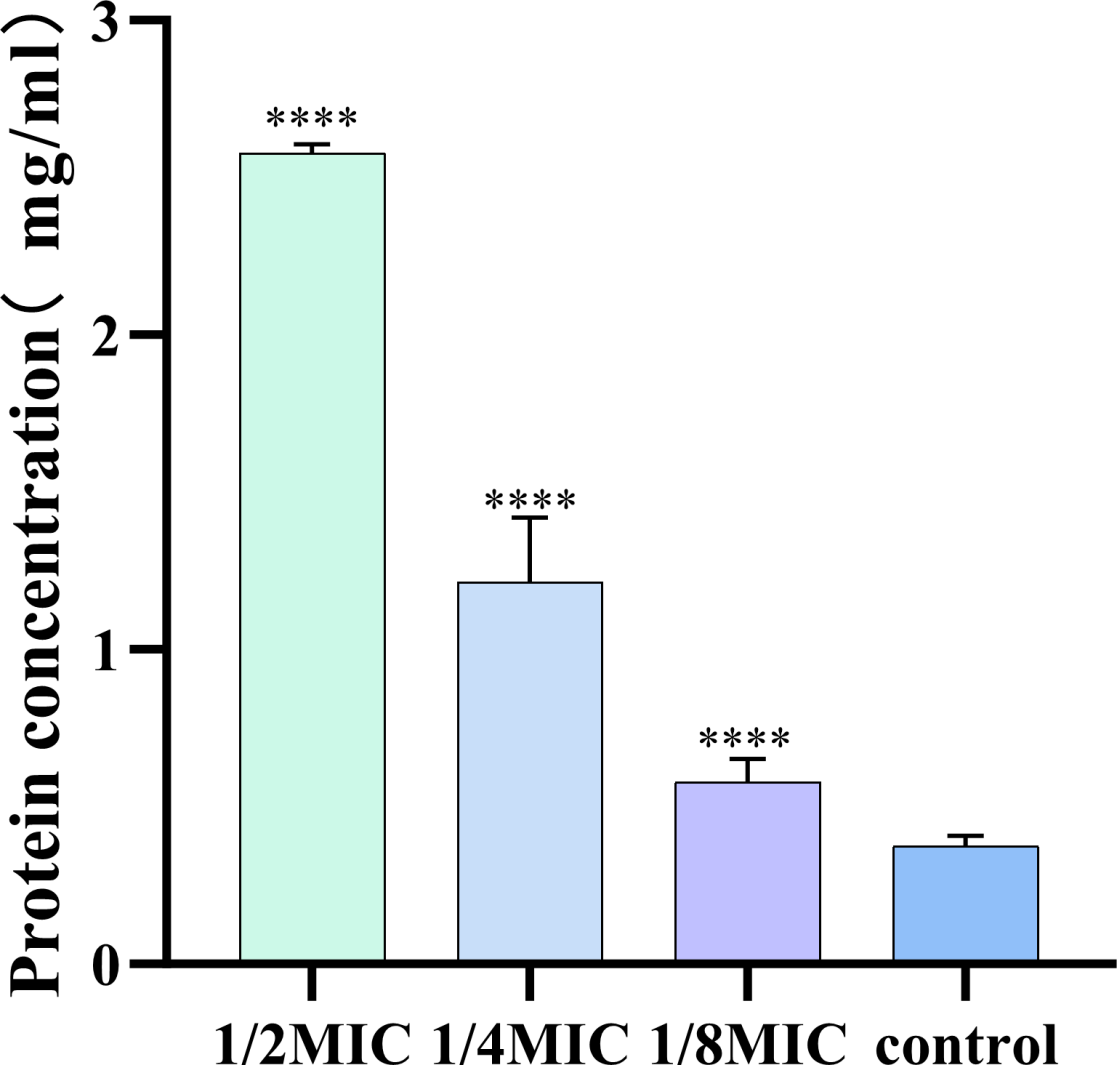
The effect of different concentrations of ICAC on extracellular proteins. The data are presented as mean ± standard deviation. ( ****P< 0.0001).

The effect of ICAC on the extracellular protein content in the biofilm of *Escherichia coli* was determined using the Bradford method. The results demonstrated that as the concentration of ICAC increased, the extracellular protein content in the biofilm of *E. coli* progressively increased (**Figure 6**). These finding suggest that ICAC disrupted the structure of the biofilm to some extent, leading to release of extracellular proteins that were originally encapsulated within the biofilm. With increasing drug concentration, the damage to the biofilm became more pronounced, resulting in a greater release of proteins into the surrounding environment.

### 3.8 Motor activity measurement

**Figure 7:**
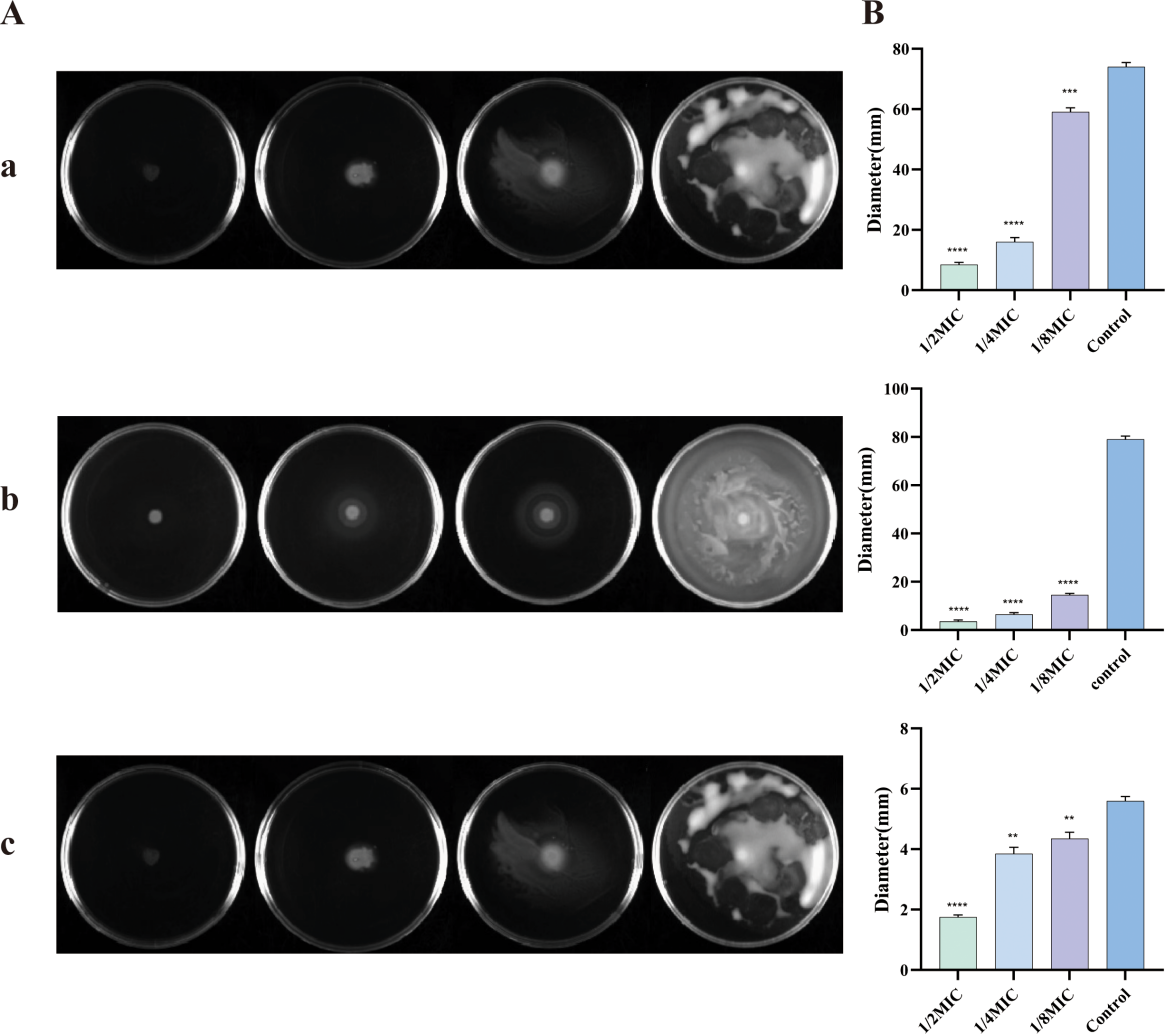
The effect of different concentrations of ICAC on *E coli* motility. **(A)** Motility images after incubation with *E coli* at different concentrations. **(B)** Quantitative assessment of motility based on halo diameter. **(a–c)** are 0.3%, 0.5%, 1% LB agar). The data are presented as mean ± standard deviation. (**P< 0.01; ***P<0.001; ****P< 0.0001).

The cyclic di-GMP system regulate the motility of *E. coli*, and ICAC was observed to inhibit the *E. coli* motility in a dose-dependently (**Figure 7A**). The diameter of the motility zone was further quantified (**Figure 7B**). From top to bottom, the images illustrate swimming motility (0.3% agar), swarming motility (0.5% agar), and twitching motility (1% agar). As the drug concentration increases, the halo diameter decreases progressively, indicating a gradual reduction in bacterial motility. These results demonstrated that ICAC significantly inhibits the motility of *E. coli* in a concentration-dependent manner.

### 3.9 Scanning electron microscopy

**Figure 8:**
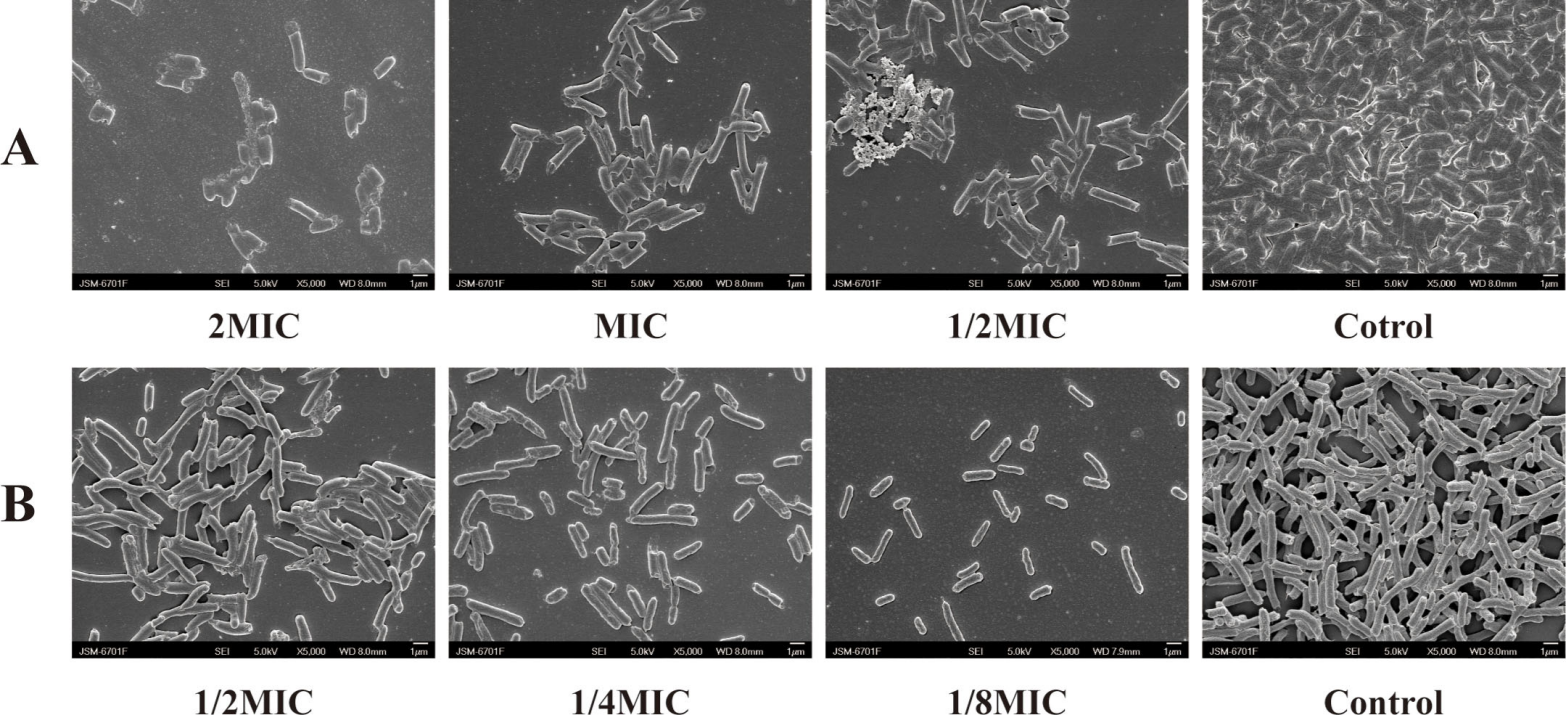
SEM images of A4E1 treated with ICAC. **(A)** Eradication; **(B)** Inhibition.

To evaluate the destructive effect of ICAC on biofilms, scanning electron microscopy (SEM) was employed to examine the morphology and structural integrity of the biofilms. As shown in **Figure 8**, in the control group, normal *E. coli* exhibited a typical rod-shaped structure with a smooth surface, and the biofilm structure remained intact. In contrast, following treatment with ICAC, the biofilm architecture was markedly disrupted, and the bacterial cells exhibited irregular shapes with increased surface roughness. Furthermore, severe cellular damage and rupture were observed, leading to compromised bacteria integrity. The surface of *E. coli* became coarse, and the cells showed evident denting and shrinkage. As the concentration of ICAC increased, the degree of biofilm destruction became more pronounced.

### 3.10 Confocal laser scanning microscope

**Figure 9:**
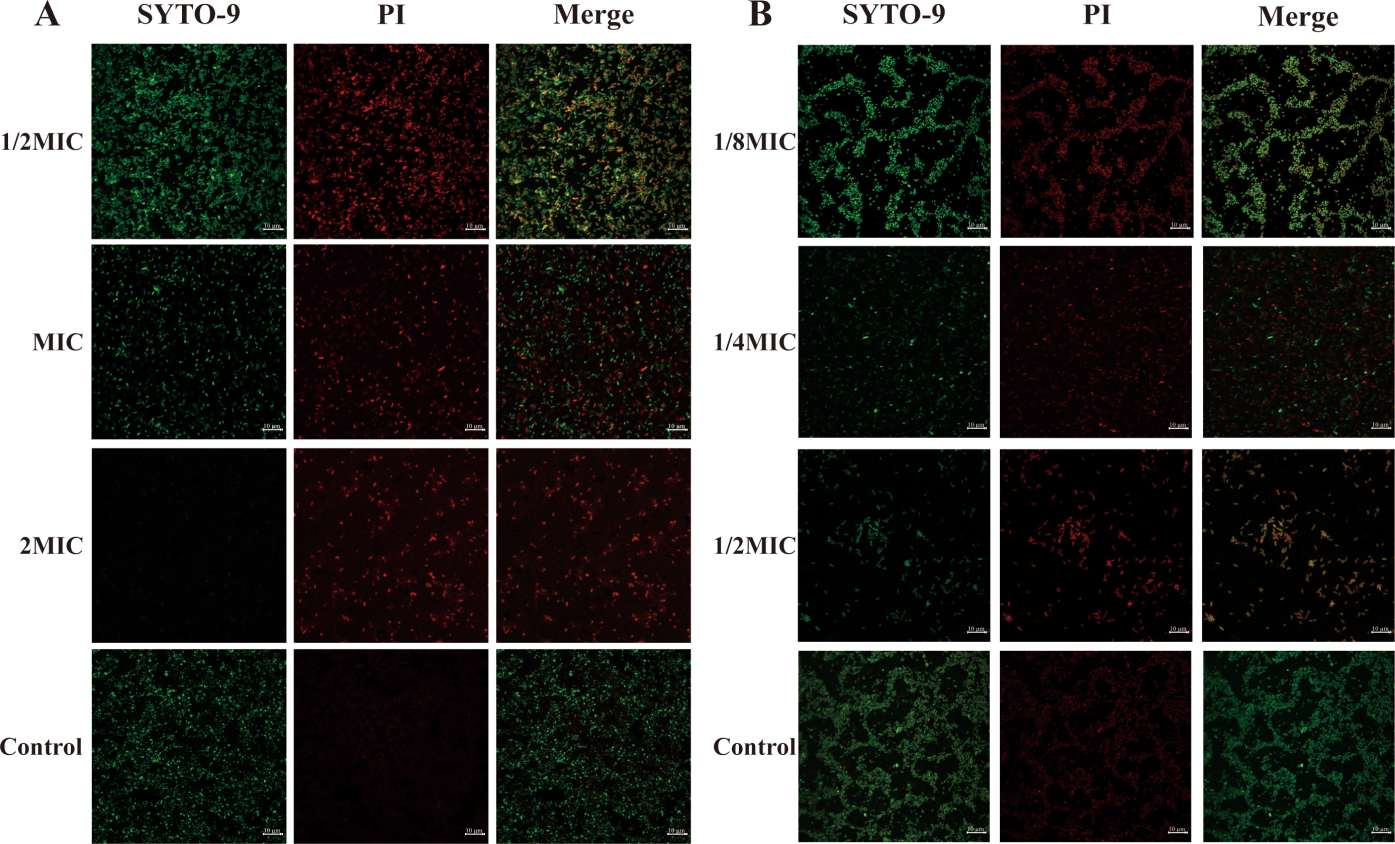
CLSM images of A4E1 treated with ICAC. **(A)** Eradication; **(B)** Inhibition.

The antibiofilm capability of ICAC was further confirmed using CLSM. As shown in  **Figure 9**, following ICAC treatment, the population of red fluorescence bacteria (dead cells) increased in response to higher drug concentration, while green fluorescence (live cells) exhibited a significant decreased. This demonstrates that ICAC effectively inhibit the adhesion of *E. coli*, reduce biofilm formation in a concentration-dependent manner, and disrupt preformed biofilms.

### 3.11 RNA isolation and qRT-PCR

**Figure 10:**
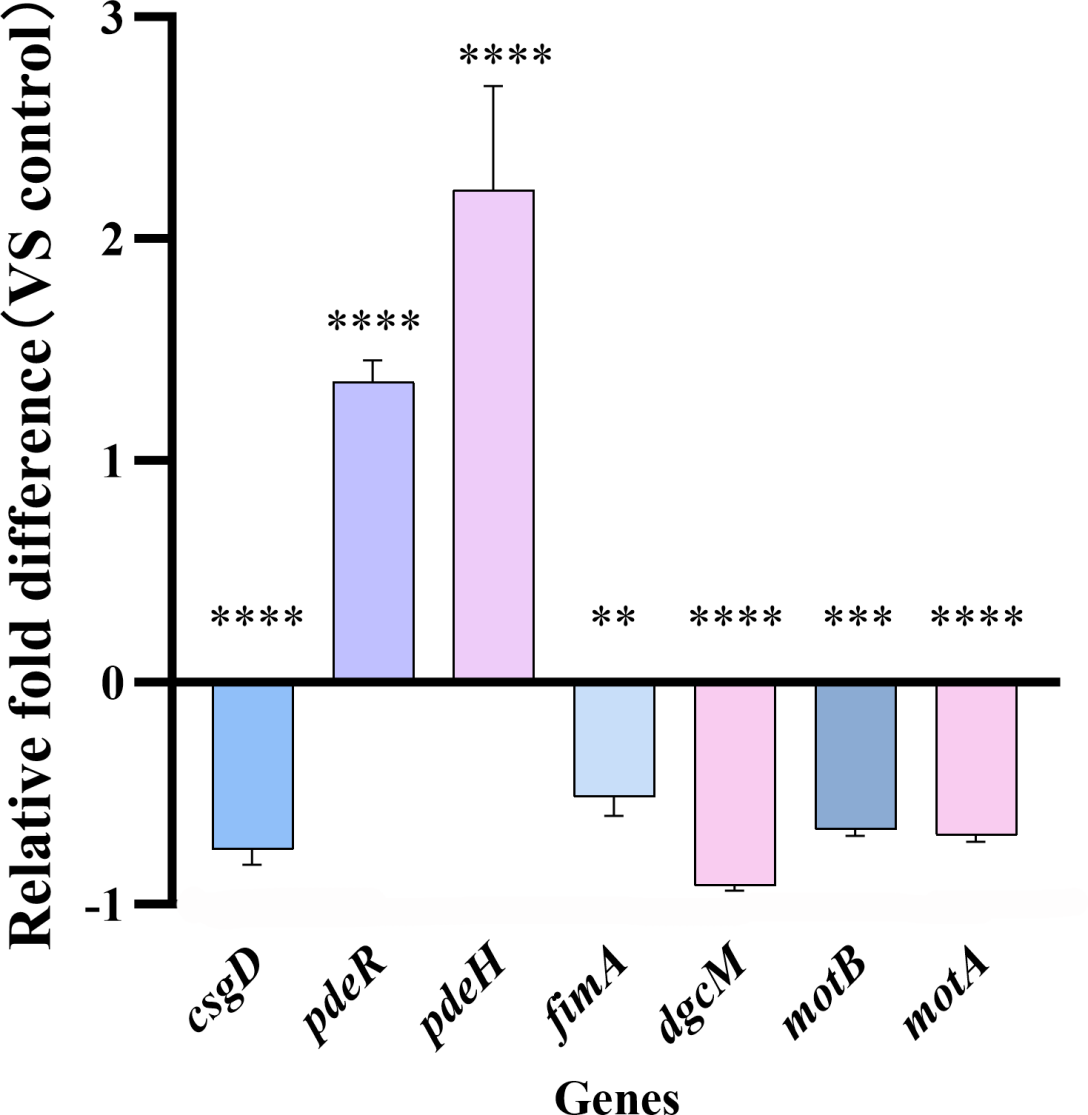
The effect of ICAC on the expression of regulatory genes in *Escherichia coli*. Data represent the mean ± standard deviation. (**P< 0.01; ***P<0.001; ****P< 0.0001).

To further investigate the potential molecular mechanism by which ICAC inhibits c-di-GMP signaling, we assessed the effects of ICAC on the expression of c-di-GMP-related, motility-related, and biofilm-related genes using qRT-PCR. Compared to the control group, ICAC downregulated the expression of the c-di-GMP synthesis gene *dgcM* (by 92%), upregulated the expression of the c-di-GMP-regulated degradation genes *pdeH* and *pdeR* (by 135% and 222%, respectively), and suppressed the expression of motility-related genes *motB*, and *motA* (by 66% and 69%, respectively), adhesion regulatory gene *fimA* (by 52%), and biofilm regulatory gene *csgD* (by 76%) (**Figure 10**). These results indicate that ICAC inhibits the expression of c-di-GMP synthesis, enhances its degradation, and consequently restricts biofilm formation and bacterial motility.

## 4 Discussion

Bacterial biofilms are ubiquitous multicellular aggregates that typically adhere to biotic or abiotic surfaces, wherein bacteria are embedded within a self-produced extracellular matrix of polymers, forming a complex and variable extracellular polymeric substance matrix (Hengge, 2019). Compared to plankton, bacteria can adapt to different environmental conditions by adjusting their biofilm structures, exhibit greater resistance to conventional antibiotic treatments and the host immune system (Mahdhi et al, 2018), enhance their tolerance to environmental stresses, and can initiate microbial infections (Condinho et al, 2023). Herein, the present study report the antifilm ang antibacterial of ICAC against *E. coli*. Furthermore, ICAC suppresses the expression of csgD, fimA, dgcM, motA, and motB genes, while enhancing the expression of pdeR and pdeH genes. Therefore, wo concluded that ICAC inhibits bacterial motility, reduces EPS production, and modulates the c-di-GMP signaling system, thereby disrupting biofilm formation in highly drug-resistant *E. coli*.

The biofilm state of pathogens promotes antibiotic resistance, thereby making bacterial infections difficult to treat (Lu et al, 2021). It has been confirmed that the biofilm formed by *coli* strains can enhance antibiotic resistance (Trang et al, 2023). In this experiment, it was also demonstrated that strong biofilm forming strains have resistance to multiple antibiotics. Antibiotics primarily target planktonic bacteria rather than biofilm bacteria, whereas natural antibiotic compounds, such as plant polyphenols, can inhibit bacterial adhesion and the development of biofilms (Stauder et al, 2010). In our study, we found that the polyphenolic compound ICAC can inhibit and eradicate biofilms in a dose-dependent manner.

Biofilm is an aggregate of microorganisms that adhered to the substrate (such as stainless, glass, meats, and vegetables) and encapsulated within a self-produced matrix composed of EPS, proteins, and extracellular DNA (eDNA) (Tuytschaever et al, 2025). EPS holds the biofilm together, protecting bacteria from predators, antibiotic chemicals, and fluid shear displacement (Jin et al, 2020). Furthermore, EPS secretion facilitates bacterial adhesion and colonization on abiotic surfaces (Vu et al, 2009). The results of this study demonstrate that ICAC can significantly reduce the adhesion of *E. coli* to polystyrene, leading to a decrease in bacterial colonization and biofilm formation in dose-dependent manner. Additionally, it is reported that ICAC effectively inhibited biomass of biofilm adhered to the cover slip surface, as observed by CLSM and SEM. In addition, Curled pili and cellulose are common EPS components in *E. coli* biofilms, whose productions is regulated by the transcriptional regulator *csgD* (Dieltjens et al, 2020). Moreover, *csgD* promotes biofilm formation by activating the expression of the csg operon, which encodes curli (Brombacher et al, 2003). *CsgD* directly suppresses the expression of flagellar genes, including fliE and the fliEFGH operon, thereby inhibiting flagellum assembly and rotation, which in turn modulates biofilm formation and bacterial motility. *MotA* and *motB* are transmembrane proteins that together form a proton channel, which couples with the proton flow. The C-terminal periplasmic domain of *motB* (motBC) binds to the peptidoglycan layer, allowing the motA/B proton channel complex to function as an active unit in flagellar motion (Kojima et al., 2009). This study showed that ICAC inhibits both EPS production and motility in a dose-dependent manner by down suppress *csgD*, *motA*, and *motB* expression. These results showed that ICAC could reduce biofilm formation by inhibiting EPS production and motility.

The cyclic diguanylate (c-di-GMP) system, as a bacterial second messenger, regulates various processes, including biofilm formation, motility, and serves as a primary regulator of host-microbe symbiosis (Zhan et al., 2024). c-di-GMP is produced by DGC and hydrolyzed by PDE. The synthesis and degradation of c-di-GMP are primarily associated with two distinct enzymes known as diguanylate cyclase (DGC) and phosphodiesterase (PDE): diguanylate cyclase (DGC), containing the GGDEF domain, synthesizes c-di-GMP from two molecules of guanosine triphosphate (GTP) and functions by binding to corresponding receptors. In this study, the expression of DGC-related genes *dgcE* and *dgcM* significantly decreased, while the expression of *pdeR* and *pdeH* significantly increased. The results indicate that ICAC may reduce c-di-GMP levels. The level of c-di-GMP plays a crucial role in controlling the transition of bacteria from motility to a sessile state. High intracellular levels of c-di-GMP are associated with biofilm formation or a sessile lifestyle, while low levels of c-di-GMP are linked to motility or a planktonic existence (Ha, 2015). In *E. coli*, deleting the DGC-encoding gene and increasing the intracellular concentration of c-di-GMP reduces bacterial swimming speed, while deleting the PDE synthesis gene and decreasing the concentration of c-di-GMP enhances bacterial swimming speed (Ko and Park, 2000). This is consistent with our results of measuring the motility of *E. coli* by ICAC. These results demonstrated that ICAC reduces biofilm formation by modulating the c-di-GMP signaling system. Wang discovered that chlorogenic acid, which has a structure similar to that of ICAC, can function as a quorum sensing (QS) inhibitor and suppress biofilm formation (Wang et al., 2023). This mechanism of action differs from that of ICAC, as previously identified by our research.

Antibiotic resistance has become a serious problem for humanity, and antibiotic treatments are often ineffective against biofilm-associated infections, as bacteria within biofilms have developed various mechanisms to resist antibiotic therapy (Panthi et al., 2024). Therefore, the development of alternative therapies is one of the major social challenges today. Anti-virulence treatment through the use of drugs that interfere with C-di-GMP in bacterial pathogens is a strategy currently under extensive research (Xu et al., 2022). This study indicates that ICAC may be an effective antibacterial compound targeting the inhibition of *E. coli* infection by C-di-GMP, thereby providing a new therapeutic direction for treating bacterial biofilm infections and effectively alleviating bacterial resistance. However, ICAC still has limitations such as low water solubility, low bioavailability in animal bodies, and low biological activity. In future research, structural modifications should be made to ICAC to address these issues, further evaluate its performance in multi species *in vivo* models, and explore its synergistic effect with antibiotics to promote the translation of ICAC into clinical applications.

## 5 Conclusion

This study evaluates the efficacy of ICAC against *E. coli* and its biofilm. Our findings reveal that ICAC inhibits bacterial motility, suppresses EPS production, and modulates the c-di-GMP signaling system, thereby disrupting biofilm formation in highly drug-resistant *E. coli*. In summary, these results demonstrate that ICAC is a potent membrane-active antibacterial agent with promising potential for treating *E. coli* infections.

## Data Availability

The original contributions presented in the study are included in the article/**Supplementary Material**. Further inquiries can be directed to the corresponding authors.
