# Supplementary material for: Study on the antibacterial effect of the new anti-biofilm inhibitor ICAC on Escherichia coli
Source: Front Cell Infect Microbiol. 2025 Dec 10;15:1710407. doi: 10.3389/fcimb.2025.1710407 (PMC12728033; doi:10.3389/fcimb.2025.1710407)
Supplement: Supplementary file 1 [file Table1.docx]

Table S1 Primer sequence.

| gene name | primer sequence | amplicon sizes |
| --- | --- | --- |
| 16s RNA-F | GTGAAGTCATGCCAGGAGCT | 99 |
| 16s RNA-R | **CGAAGTATGCGTCCGGATCA** |  |
| csgD-F | TGCAACCCATTGACAACACG | 82 |
| csgD-R | ACCGCGACATTGAAAACTGG |  |
| pdeR-F | CGGCTCCCCTTTCGCATTGG | 149 |
| pdeR-R | TGTCCTCGTCCGCCTTCCTTC |  |
| pdeH-F | TTCAGAACTCGCCCGCATTCG | 86 |
| pdeH-R | CGCCAGAACCGCCGTATTCAG |  |
| fimA-F | TTGCGATACCAATGTTGCAT | 194 |
| fimA-R | TCAGGGTTGTTTGCTCACTG |  |
| dgcM-F | CATCATTTTTCGCCCGATAC | 190 |
| dgcM-R | CTACCAAGAACCCCACTGGA |  |
| motB-F | CAGTGCCGATGTCGAACCCTATATG | 146 |
| motB-R | GCTCCCAGTTGCTATATCCTTTCTCAC |  |
| motA-F | GAAGCCTTGGAGCACTCTATCAACC | 150 |
| motA-R | CTTTGGTGTATTTGGAGCGACGAAAC |  |

Tabse S2 Determination of biofilm formation ability of *E. coli*

| biofilm-forming ability | Strains | OD570 | | |
| --- | --- | --- | --- | --- |
| Strong biofilm-forming ability | C3E2 | 0.612 | 0.609 | 0.548 |
|  | C4E2 | 0.495 | 0.523 | 0.489 |
|  | B1P1 | 0.661 | 0.655 | 0.598 |
|  | A4E1 | 0.756 | 0.735 | 0.689 |
|  | B6P1-1 | 0.459 | 0.558 | 0.581 |
|  | C5E1-1 | 0.626 | 0.557 | 0.542 |
|  | C1E2 | 0.389 | 0.452 | 0.415 |
|  | B6P2-2 | 0.435 | 0.458 | 0.487 |
|  | C5E1 | 0.536 | 0.529 | 0.539 |
| Moderate biofilm-forming ability | C4E1 | 0.224 | 0.296 | 0.262 |
|  | 1G2 | 0.227 | 0.281 | 0.265 |
|  | B3E1 | 0.352 | 0.348 | 0.349 |
|  | F2E1-2 | 0.333 | 0.314 | 0.289 |
|  | C6P1-2 | 0.402 | 0.392 | 0.308 |
|  | A6P1-1 | 0.285 | 0.266 | 0.274 |
|  | C6P1-1 | 0.296 | 0.287 | 0.288 |
|  | 3B | 0.369 | 0.357 | 0.361 |
|  | C1E1 | 0.247 | 0.223 | 0.236 |
|  | B6E2 | 0.286 | 0.274 | 0.286 |
|  | B1P2 | 0.325 | 0.318 | 0.336 |
|  | F2E1-1 | 0.326 | 0.338 | 0.328 |
|  | H2-2 | 0.254 | 0.259 | 0.249 |
|  | H2-1 | 0.315 | 0.307 | 0.311 |
|  | D5E2-2 | 0.258 | 0.269 | 0.257 |
|  | C6E1 | 0.366 | 0.347 | 0.295 |
| Weak biofilm-forming ability | A10E4-1 | 0.172 | 0.177 | 0.191 |
|  | A4E1 | 0.201 | 0.205 | 0.177 |
|  | A6E1 | 0.188 | 0.188 | 0.145 |
|  | D5E1 | 0.194 | 0.199 | 0.172 |
|  | B4E1-2 | 0.176 | 0.262 | 0.165 |
|  | A5P1-2 | 0.189 | 0.183 | 0.221 |
|  | A5E1-1 | 0.268 | 0.161 | 0.180 |
|  | A10E2 | 0.187 | 0.132 | 0.161 |
|  | C5E2 | 0.209 | 0.187 | 0.179 |
|  | 7E1-2 | 0.184 | 0.125 | 0.188 |
|  | C1E1 | 0.207 | 0.149 | 0.202 |
|  | A10E3 | 0.148 | 0.131 | 0.177 |
|  | B3E1-2 | 0.193 | 0.121 | 0.184 |
|  | B3E1-1 | 0.198 | 0.135 | 0.190 |
|  | B4E1-3 | 0.142 | 0.249 | 0.124 |
|  | 9E1-1 | 0.178 | 0.376 | 0.168 |
|  | 3G1 | 0.179 | 0.217 | 0.157 |
|  | A4P2 | 0.193 | 0.243 | 0.159 |
|  | C6P1-1 | 0.233 | 0.206 | 0.166 |
|  | A10E4-1 | 0.217 | 0.195 | 0.112 |
|  | 4E1-2 | 0.224 | 0.180 | 0.128 |
|  | 14E3 | 0.236 | 0.167 | 0.166 |
|  | H6-1 | 0.176 | 0.287 | 0.218 |
|  | A5P2 | 0.199 | 0.184 | 0.161 |
|  | B10E1 | 0.198 | 0.157 | 0.202 |


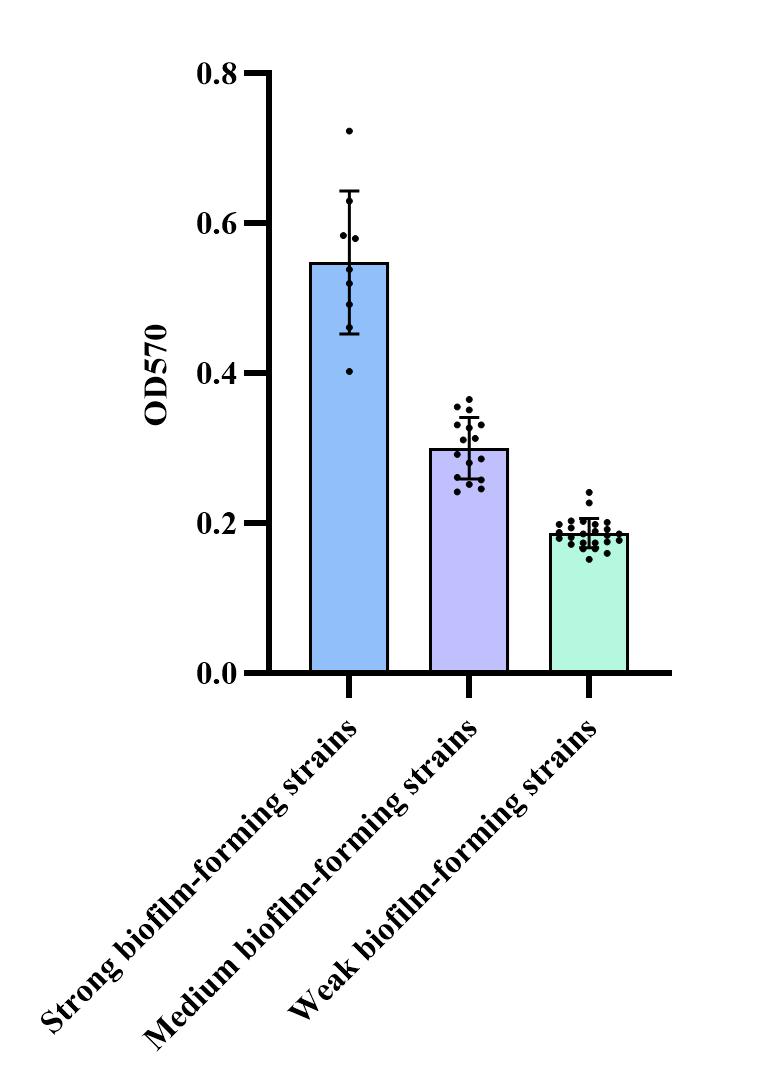


Figure S1 Determination of biofilm-forming ability in biofilm-positive strains

Table S3 Antimicrobial Susceptibility Table.

| Strain name | | Ampicillin | Cefotaxime | Aztreonam | Meropenem | Tetracycline | Doxycycline | Tigecycline | Ciprofloxacin |
| --- | --- | --- | --- | --- | --- | --- | --- | --- | --- |
| C3E2 | S | | R | R | R | S | S | R | S |
| C4E2 | R | | S | R | R | R | S | R | S |
| B1P1 | R | | S | R | R | S | S | S | R |
| A4E1 | R | | R | R | R | R | S | R | R |
| B6P1-1 | R | | R | S | S | S | R | R | R |
| C5E1-1 | R | | S | R | R | R | R | R | S |
| C1E2 | S | | R | R | R | S | S | S | R |
| B6P2-2 | S | | S | R | R | R | S | S | R |
| C5E1 | R | | R | R | R | S | S | S | S |
